# Retinoic acid induces HL-60 cell differentiation via the upregulation of miR-663

**DOI:** 10.1186/1756-8722-4-20

**Published:** 2011-04-25

**Authors:** Pan Jian, Zhao Wen Li, Tao Yan Fang, Wang Jian, Zhou Zhuan, Liao Xin Mei, Wu Shui Yan, Ni Jian

**Affiliations:** 1 Department of Hematology and Oncology, Children's Hospital of Soochow University, Suzhou, China; 2 Hillman Cancer Center Lab, Department of Pathology, Pittsburgh University, G21 5117 Centre Ave. Pittsburgh, PA 15206 USA; 3 Translational Research Center, Second Hospital, The Second Clinical School, Nanjing Medical University, Nanjing, China

## Abstract

**Background:**

Differentiation of the acute myeloid leukemia (AML) cell line HL-60 can be induced by all trans-retinoic acid (ATRA); however, the mechanism regulating this process has not been fully characterized.

**Methods:**

Using bioinformatics and *in vitro *experiments, we identified the microRNA gene expression profile of HL-60 cells during ATRA induced granulocytic differentiation.

**Results:**

Six microRNAs were upregulated by ATRA treatment, miR-663, miR-494, miR-145, miR-22, miR-363* and miR-223; and three microRNAs were downregulated, miR-10a, miR-181 and miR-612. Additionally, miR-663 expression was regulated by ATRA. We used a lentivirus (LV) backbone incorporating the spleen focus forming virus (SFFV-F) promoter to drive miR-663 expression, as the CMV (Cytomegalovirus) promoter is ineffective in some lymphocyte cells. Transfection of LV-miR-663 induced significant HL-60 cell differentiation *in vitro*.

**Conclusions:**

Our results show miR-663 may play an important role in ATRA induced HL-60 cell differentiation. Lentivirus delivery of miR-663 could potentially be used directly as an anticancer treatment in hematological malignancies

## Background

Differentiation of the acute myeloid leukemia (AML) cell line HL-60 can be induced by all trans-retinoic acid (ATRA); however, the mechanism regulating this process is not yet fully understood [1]. Erkel et al. reported that growth arrest and induction of differentiation of HL-60 cells in response to Sch 52900 is due to induction of the cell cycle inhibitor p21WAF, and inhibition of the extracellular signal-regulated kinase (ERK) signaling pathway, leading to activation of the transcription factor AP-1 [2]. Microarray analysis has shown ATRA can induce upregulation of genes involved in differentiation, the oxidase activation pathway and adhesion molecules. In HL-60 cells, ATRA treatment induces differential expression of a variety of genes from several pathways, including the differentiation pathway [3-5]. So far, few studies have focused on expression of microRNAs during HL-60 differentiation, and the expression profiles of human miRNAs during cell differentiation remain largely unknown.

This study analyzed the microRNA expression profile in HL-60 cells treated with ATRA. to investigate whether ATRA can induce growth arrest via upregulation of miR-663 expression, which has been linked to modulation of the cell cycle and mitotic growth arrest [6]. Our results showed both ATRA and miR-663 can significantly inhibit HL-60 cell proliferation and induce differentiation.

MicroRNAs regulate the expression of genes involved in the control of development, proliferation, apoptosis, and stress responses [7-9]. Analysis of microRNA expression and function during hematopoiesis has unraveled the existence of several complex regulatory loops by which microRNAs fine-tune hematopoietic differentiation and proliferation. The expression profiles of miR-142 [10,11], miR-181 [12-14] and miR-223 [14-16] have been described in B cells, T cells, monocytes, granulocytes and erythroid cells in murine hematopoiesis. Ectopic expression of these miRNAs dramatically alters the proportion of differentiated murine hematopoietic cell lineages *in vitro *and *in vivo *[17-21]. This suggests miRNAs can play an important lineage-specific role in mammalian cell differentiation. In humans, miR-107 and miR-223 are upregulated during ATRA induced granulocytic differentiation. Both miR-107 and miR-223 are postulated to downregulate their target gene NFI-A, and mediate a regulatory loop during cell differentiation [22]. This data suggests miRNAs can function as oncogenes or tumor suppressors, and play important roles in the genesis of leukemia. Using a bioinformatic approach followed by *in vitro *experiments, we identified the microRNA gene expression profile of the AML cell line HL-60 during ATRA induced granulocytic differentiation. We further demonstrated that miR-663 expression level is regulated by ATRA.

Lentiviral technology represents a powerful method to genetically modify leukemia cells. We chose to use a viral expression backbone driven by the spleen focus forming virus (SFFV-F) promoter, as the CMV (Cytomegalovirus) promoter has been shown to be ineffective in some lymphocyte cells. We focused on miR-663 as a candidate molecule which is important for HL-60 cell differentiation. The virus expressing the miR-663 precursor was compared to a control mock virus containing GFP. Lentivirus transfection showed LV-miR-663 significantly induces HL-60 cell differentiation *in vitro*. miR-663 may play important role in the differentiation of HL-60 cells treated with ATRA and miR-663 lentivirus could potentially be used directly as an anticancer treatment in he

## Methods

### 2.1 Cell line and reagents

HL-60 cells were obtained from our own laboratory. ATRA, RPMI 1640, MTT, DMSO, TPA and NBT were obtained from Sigma Co. DMEM was obtained from Invitrogen. PCR primers were synthesized by Shanghai Sangon Biotechnology Co. Ltd. PE-conjugated CD11b (ITGAM integrin alpha M) antibody was purchased from Pharmingen Co.

### 2.2 Cell culture and induction

HL-60 cells were cultured in RPMI 1640 standard medium with 2 mmol/L L-glutamine supplemented with 10% heat-inactivated fetal calf serum, 100 U/ml penicillin and 100 μg/ml streptomycin at 37°C in 5% CO_2_. Exponentially growing cells (approximately 1 × 10^7^) were incubated with 0.1 μmol/L ATRA, 0.1% alcohol or untreated RPMI 1640 for 1 to 3 days.

### 2.3 MTT proliferation assay

Cell proliferation was determined using the MTT (methyl thiazolyl tetrazolium) assay. HL-60 cells (5 × 10^5^/well in 96-well plates) were incubated with 0.1 μmol/L ATRA [23], 0.1% alcohol or untreated RPMI 1640 for 24 to 72 h, then 10 μl 5 mg/m1 MTT was added to each well for 4 h. The reaction was stopped by addition of 150 μl DMSO and absorbance (A) at 490 nm was determined on a plate reader (Bio-Rad). Each group was analyzed in triplicate samples. Cell inhibition rate = 100% × (control group A values -experimental group A values)/control group A values.

### 2.4 NBT and CD11b differentiation assays

Differentiation of HL-60 cells was assessed using the NBT (nitroblue tetrazolium) reduction test and flow cytometry detection of the cellular surface differential antigen CD11b. Briefly, 100 μl 1 × 10^6^/m1 HL-60 cells in 96-well plates were incubated with 0.1 μ mol/L ATRA [23] for 1 to 3 days. RPMI 1640 was used as a blank control and 0.1% alcohol was used as the solvent control. 100 μl 1 mg/ml NBT and 200 μl 1 mg/ml TPA were added to each well and incubated at 37°C in 5% CO_2 _for 1 h, after which the cells were centrifuged for 5 min and then subjected to Wright's staining. When NBT is phagosomed by cells, the intracellular dye converts to insoluble blue formazan crystals [24]. The number of positive cells containing blue formazan crystals was determined from two hundred cells using microscopy with an oil immersion objective. For detection of the cell differentiation antigen CD11b [25,26], 1 × 10^6 ^cells were washed twice with PBS, incubated with PE-conjugated CD11b antibody or PE-conjugated IgG1isotype control antibody at 4°C for 30 min and analyzed by flow cytometry using a FACScan flow cytometer and Cell Quest software (Becton Dickinson, Mountain View, CA). The expression rate of CD11b positive cells was determined from 1 × 10^4 ^cells for each group.

### 2.5 MicroRNA expression profiling

MicroRNAs were extracted using the mirVana miRNA isolation kit (AM1560, Applied Biosystems, USA). Samples which were successfully isolated were analyzed using an Agilent miRNA Chip version 10.0 at the Microarray Core Facility, Baylor College of Medicine, USA. In total, 637 images were acquired, calculated, normalized and filtering of signal intensity for each spot and batch-effect adjustment was performed. A total of 235 microRNA probes met the filtering criteria for subsequent analysis using significance analysis of microarrays (SAM, Version 3.0, 2007, http://www-stat.stanford.edu/~tibs/SAM/).

### 2.6 miRNA extraction and real-time quantitative PCR (qRT-PCR) assays

Extraction of miRNA was performed using the mirVana miRNA isolation kit and TaqMan miRNA assays were used to detect and quantify mature miR-663 as previously described [6]. Briefly, total RNA was reverse transcribed using the Reverse Transcription Kit (Applied Biosystems Inc., CA), according to the manufacturer's instructions. The RT primers were: U6 5'-CGCTTCACGAATTTGCGTGTCAT-3' and mir-663 5'-GTCGTATCCAGTGCGTGTCGTGGAGTCGGCAATTGCACTGGATACGACGCGGTCC-3'. The PCR primers used to quantify U6 expression were:

F: 5'-GCTTCGGCAGCACATATACTAAAAT-3' and R: 5'-CGCTTCACGAATTTGCGTGTCAT-3' and for mir-663 were: F: 5'-GTGCGTGTCGTGGAGTCG-3' and R: 5'-TTTAGGCGGGGCG-3'. mir-663 expression was normalized to endogenous U6 expression using the SDS relative quantification software (Applied Biosystems Inc, USA).

### 2.7 MicroRNA lentiviral expression constructs and lentivirus production

The lentiviral vector expressing miR-663 has been previously described [6]. Briefly, an approximately 250 bp fragment containing the human miR-663 precursor hairpin loops was amplified by PCR using using primers flanked by *BamH*I and *Xho*I sites at the 5' and 3' ends, and cloned into the pDrive cloning vector (Qiagen) under the control of the RNA Pol III mouse U6 promoter. Positive clones were confirmed by sequencing and subcloned into the pHR' SINcPPT SFFV-WPRE vector under the control of the SFFV promoter. The GFP virus, driven by the SFFV promoter, has also been previously described [27]. The vector plasmids, gag-pol plasmid (pD8.91) and the VSVG envelope encoding plasmid (pMD2-G), were amplified in *E.Coli *and purified using the Endofree Maxiprep Kit (Qiagen). 13 μg transfer vector, 10 μg pD8.91 and 6 μg pMD2-G was mixed with 1.5 mL 0.25 M CaCl_2 _(Sigma) and added to 1.5 mL 2 × HEPES (Sigma) and mixed while bubbling for 20 min to allow a precipitate to form. This was then added to a 175 cm^2 ^flask of approximately 60% confluent 293T cells containing 20 mL DMEM supplemented with 10% fetal calf serum, 100 U/mL penicillin, 100 μg/mL streptomycin and 2 mM glutamine and incubated for 48 h at 37°C in 5% CO2. The supernatant was centrifuged at 1,700 g for 10 min to pellet cell debris, and ultracentrifuged at 121,603 g for 2 h. The pellet containing concentrated virus was resuspended in DMEM without supplements and stored at -80°C.

### 2.8 Statistical analysis

All data are presented as mean ± SD. Statistical analysis was performed using SPSS (Chicago, IL). Student's two-tailed *t*-tests were used to compare groups and *p *≤ 0.05 was considered significant.

## Results and discussion

**Figure 1:**
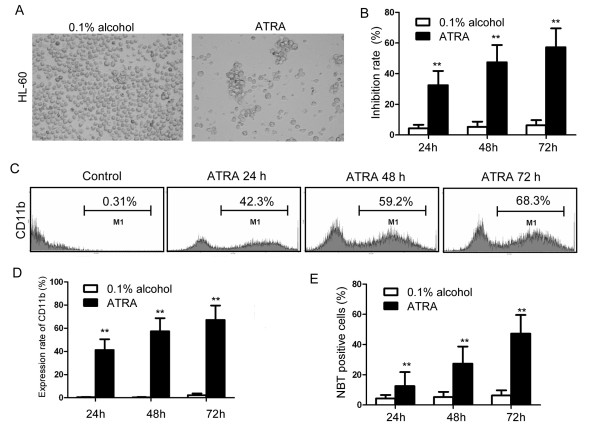
**ATRA inhibits proliferation and induces differentiation in HL-60 cells**. (A) Morphology of HL-60 cells treated with 0.1 μmol/L ATRA or 0.1% alcohol at 48 hours. (B) Cell inhibition rates in ATRA and 0.1% alcohol treated HL-60 cells at 24-72 hours, determined using the MTT assay. Each group was assayed in triplicate. Cell inhibition rate was calculated as 100% × (control group A values -experimental group A values)/control group A values. (C-E) HL-60 cell differentiation was assessed using CD11b and the NBT reduction test. (C-D) CD11b flow cytometry analysis of cells treated with ATRA or 0.1% alcohol. The expression rate was determined as the number of CD11b positive cells in 1 × 10^4 ^cells. (E) NBT analysis of HL-60 cells treated with ATRA or 0.1% alcohol. Two hundred cells were observed and positive cells with blue formazan crystals were counted by microscopy, **p < 0.01.

ATRA inhibited HL-60 cell proliferation (Figure 1A and 1B). After incubation with 0.1 μmol/L ATRA, the inhibition rates of HL-60 cells determined using the MTT assay were 32.5 ± 9.3%, 47.4 ± 11.3% and 57.2 ± 12.4% at 1, 2 or 3 days respectively, compared with the solvent control group, *p *< 0.01. These results indicate ATRA can inhibit HL-60 proliferation in a time-dependent manner.

ATRA also induced HL-60 mature granulocyte cell differentiation (Figures 1C and 1D). Treatment with ATRA for 1 to 3 days significantly increased the number of HL-60 cells expressing CD11b. After 1, 2 and 3 days the expression rates of CD11b in ATRA treated cells were 41.2 ± 9.1%, 57.4 ± 11.4% and 67.2% ± 12.4% respectively, compared with the solvent control group (0.56 ± 0.21%, *p *< 0.01). The percentage of NBT positive cells in HL-60 cells treated with ATRA for 1, 2 or 3 days (Figure 1E) was 12.5%±9.1%, 27.4% ± 10.3% and 47.2% ± 10.4% respectively, compared with the solvent control group 4.31% ± 2.3%, *p *< 0.01, providing further evidence that ATRA promotes HL-60 cell differentiation.

**Figure 2:**
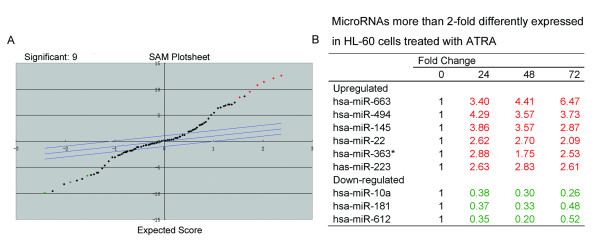
**MicroRNA expression in HL-60 cells during ATRA-induced differentiation**. The microRNA profile in HL-60 cells treated with ATRA or 0.1% alcohol was determined using a microRNA microarray as described in the materials and methods (A) significance analysis of microarrays of differentially regulated microRNAs in ATRA treated cells. (B) List of differentially expressed microRNA and their fold expression changes.

A miRNA microarray identified the expression of several microRNAs significantly changed in HL-60 cells during ATRA-induced differentiation. Significance analysis of microarrays (Figure 2A) was used to identify miRNAs whose expression was altered more than 2 fold in response to treatment with ATRA for 24-72 h, and the differentially expressed microRNAs are listed in Figure 2B.

**Figure 3:**
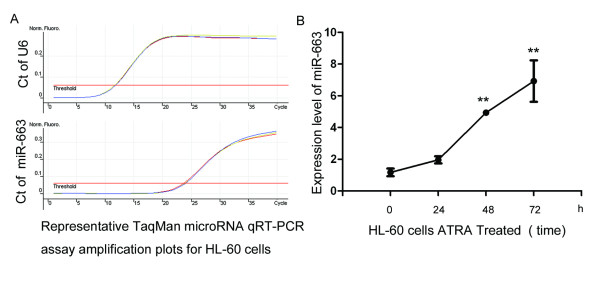
**ATRA treatment significantly upregulates miR-663 in HL-60 cells**. (A) TaqMan qRT-PCR miRNA assays were used to quantify the time course of mature miR-663 expression in HL-60 cells treated with ATRA. Expression was normalized to endogenous U6 expression. (B) Summary of TaqMan qRT-PCR miRNA assay results showing miR-663 was significantly upregulated in ATRA treated cells. At 72 h, mir-633 expression in ATRA treated cells (6.93 ± 1.31) was significantly increased compared to cells treated with 1% ethanol (1.17 ± 0.24, *p *< 0.01).

We confirmed miR-663 was significantly upregulated by ATRA treatment using TaqMan mircoRNA qRT-PCR assays. MiR-663 is a challenging molecule to amplify using PCR as the microRNA precursor consists of a highly stable hairpin due to GC base paring; however, novel technologies have been developed to successfully amplify and quantify the mature miR-663. Real-time PCR has become the gold standard of nucleic acid quantification due the high specificity and sensitivity and technological advancements have enabled quantification of microRNAs in a comparable manner to mRNAs. The time course of mature miR-663 expression determined by qRT-RCR (Figure 3A) indicated miR-663 was significantly upregulated in ATRA treated HL-60 cells. After 72 h, expression of miR-633 in the ATRA treated group was 6.93 ± 1.31 compared with the control group 1.17 ± 0.24, Figure 3B, *p *< 0.01.

**Figure 4:**
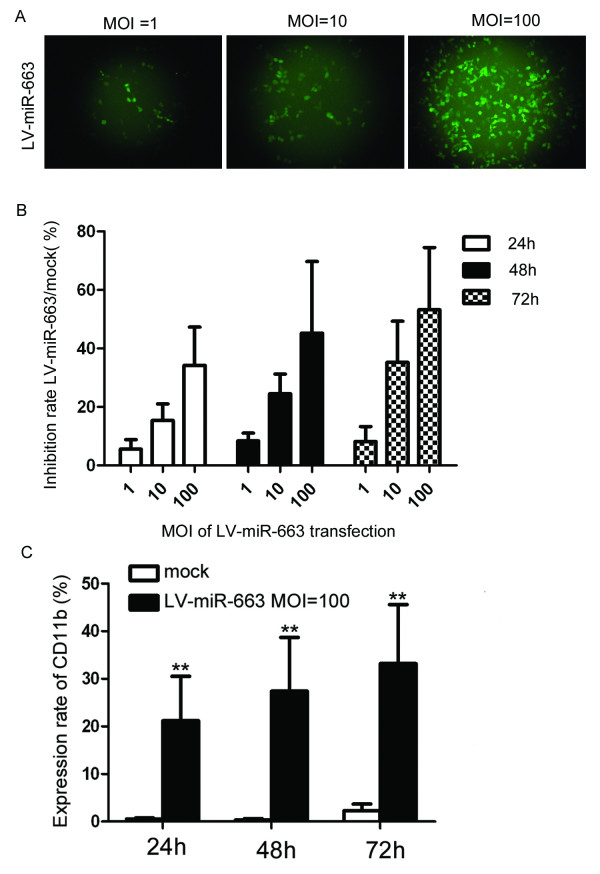
**Lentivirus expressing miR-663 induces HL-60 cell differentiation**. A lentivirus miR663 expressing (LV-miR-663) construct was generated. (A) Multiplicities of infection (MOI) of 1, 10 and 100 were used to determine optimal transduction efficiency in HL-60 cells using a control GFP-expressing lentivirus; GFP was detected in approximately 80% cells in the MOI 100 group. (B) MTT assays indicated the inhibition rates of HL-60 transfected with LV-miR-663 were 34.2 ± 13.1%, 45.2 ± 24.5% and 53.2 ± 21.3% at 1, 2 and 3 days, respectively, compared with mock transfected cells, *p *< 0.01. These results indicate miR-663 expression inhibits HL-60 proliferation in a time-dependent manner. (C) miR-663 induces HL-60 differentiation to mature granulocytes. In cells transfected with LV-miR-663 expression of the differentiation marker CD11b was increased significantly and after 1, 2 or 3 days the expression rates of CD11b were 21.2 ± 9.3%, 27.4 ± 12.5% and 33.2% ± 12.4% respectively in LV-miR-663 transfected cells, compared with the mock transfected cells (0.56 ± 0.21%, *p *< 0.01).

Recombinant vectors based on retroviruses, including both onco-retroviruses and lentiviruses, remain the only choice to efficiently and stably transduce leukemia cells. Lentiviruses (LV) offer several advantages. Firstly, LV can transduce both dividing and non-dividing cells including freshly isolated hematopoietic stem cells and T cells in blood. Secondly, LV can accommodate various transcriptional promoters, either ubiquitous or cell-specific; and thirdly, self-inactivating safety modifications, which permanently disable viral promoters within the viral long-terminal repeat region after integration, enables control of transgene expression in the targeted cells solely by internal promoters. We used a SFFV promoter lentiviral backbone as the CMV promoter is ineffective in some lymphocyte cells. When transducing a lentiviral construct into a cell line for the first time, a range of volume or MOI (multiplicities of infection) should be tested. MOIs of 1, 10 and 100 were used to determine the optimal transduction efficiency using a control plasmid. High transduction efficiency was observed in the MOI 100 group, where approximately 80% of the cells expressed GFP (Figure 4A).

Transfection of the miR-663 lentivirus, LV-miR-663, inhibited HL-60 proliferation in a time-dependent manner. MTT assays indicated inhibition rates were 34.2 ± 13.1%, 45.2 ± 24.5% and 53.2 ± 21.3% at 1, 2 and 3 days respectively, compared with the mock transfected group, *p *< 0.01, Figure 4B. LV-miR-663 also induced HL-60 differentiation and lead to a significant increase in the rate of CD11b expression, indicating mature granulocyte differentiation.

ATRA is the acid form of vitamin A, and can inhibit proliferation and induce differentiation in tumor cells. As a physiological inducer of differentiation, ATRA has been successfully applied in the treatment of hematological malignancies and has become a model of differentiation therapy (8). It has been demonstrated that PML-RARa is able to influence transcription of several miRNA genes [10,13]. As the expression of these miRNAs is restored by ATRA, our results suggest the effects of successful clinical protocols to eradicate APL cells may be mediated, in part, by affecting microRNA expression. These findings also indicate that ATRA may also indirectly affect gene transcription through the ability of microRNAs to regulate of post-transcriptional mRNA processing.

In the present study, we characterized the expression profile of microRNAs during HL-60 ATRA-induced granulocytic differentiation, and identified a small number of microRNAs upregulated and downregulated in a time-dependent manner. Our findings are consistent with the previous observations of Croce CM, Norrild B and Barrera G [28-30]. We also observed that miR-663 is upregulated in response to ATRA treatment in HL-60 cells, which is the first report of the involvement of miR-663 in ATRA-induced differentiation. In HL-60 cells, Pizzimenti et al. reported miR-663 was upregulated by 4-Hydroxynonenal (HNE) treatment [30] and Kasashima et al. reported miR-663 was upregulated during 12-O-tetradecanoylphorbol-13-acetate (TPA) induced differentiation [31]. Lutherburrow et al. reported expression of miR-663 is higher in M1 than M5 AML patients and hypothesized it may potentially be involved in blocking the differentiation of M1 blasts, and consequently monocytic differentiation [32].

MiR-663 seems to have dual functions, and the role it mediates varies in different experimental models. In human THP-1 monocytic cells and human blood monocytes, resveratrol upregulates miR-663 expression [28]. MiR-663 is an oscillatory shear (OS) sensitive microRNA, and plays a key role in OS-induced inflammatory responses by mediating the expression of inflammatory genes in HUVECs [33]. Downregulation of miR-663 in tumor cells may contribute to aberrant cell hyperplasia, leading to the development of gastric cancer [6].

Thousands of miR-663 target genes have been predicted by bioinformatic analysis and interestingly, most are transcription factors of AP-1 [28]. In THP-1 cells, miR-663 decreases endogenous activator protein-1 (AP-1) activity and impairs lipopolysaccharide (LPS) induced upregulation of AP-1 by, in part, by directly targeting the Jun B and Jun D transcripts. Dose dependent downregulation of AP-1 activity and Jun B levels by resveratrol are miR-663 dependent. The specific targeting of genes encoding a subset of AP-1 factors by mir-633, such as Jun B and Jun D, may possibly explain some of the anti-leukemia function of ATRA. Bioinformatic tools have predicted TGF-β is also a target gene of miR-663, which is of interest as TBF-β is an important molecule with roles in many signaling pathways. These findings indicate miR-663 expression is upregulated during ATRA-induced differentiation, and lentivirus expressing miR-663 can significantly induce HL-60 differentiation. This study demonstrates miR-663 may play an important role in ATRA-induced differentiation in HL-60 cells; however, the function of miR-663 and the mechanism by which it affects HL-60 differentiation requires further study.

## Conclusion

Our study is the first investigation of the effect of ATRA on microRNA expression, specifically the ability of ATRA treatment to upregulate miR-663 expression and lentiviral delivery of miR-663 can induce differentiation and inhibit proliferation in HL-60 cells.

## List of abbreviations used

AP-1: activator protein-1; AML: acute myeloid leukemia; ATRA: all trans-retinoic acid; CMV: Cytomegalovirus; ERK: extracellular signal-regulated kinase; HNE: 4-Hydroxynonenal; LPS: lipopolysaccharide; LV: Lentiviruses; MTT: methyl thiazolyl tetrazolium; NBT: nitroblue tetrazolium; OS: oscillatory shear; SFFV: spleen focus forming virus.

## Competing interests

The authors declare that they have no competing interests.

## Authors' contributions

PJ designed the study and wrote the manuscript, NJ and ZWL participated in data analysis, WJ and TYF performed RT-PCR analysis and differentiation analysis of HL-60 cells, ZZ LXM and WSY performed flow cytometry analysis. All authors read and approved the final manuscript.

## Authors' information

Pan Jian, Ph.D. Immulogy. Graduated from State Key Laboratory of Molecular Oncology, Cancer Institute (Hospital), Peking Union Medical College, Chinese Academy of Medical Sciences, Beijing, PR China. Now is an associate professor of Department of Hematology and Oncology, Children's Hospital of Soochow University, Suzhou China, and an guest professor of Translational research center, Second Hospital, The Second Clinical School, Nanjing Medical University, Nanjing, China.
